# Functional characterisation of the regulation of CAAT enhancer binding protein alpha by GSK-3 phosphorylation of Threonines 222/226

**DOI:** 10.1186/1471-2199-7-14

**Published:** 2006-04-06

**Authors:** H-K Liu, S Perrier, C Lipina, D Finlay, H McLauchlan, CJ Hastie, HS Hundal, C Sutherland

**Affiliations:** 1 Division of Molecular Physiology, School of Life Sciences, University of Dundee, Dundee, DD1 4HN, UK; 2 Division of Pathology and Neurosciences, Ninewells Medical School, University of Dundee, Dundee, DD1 9SY, UK; 3 Division of Signal Transduction and Therapy, School of Life Sciences, University of Dundee, Dundee, DD1 4HN, UK; 4 National Research Institute of Chinese Medicine, Taipei, Taiwan, Republic of China

## Abstract

**Background:**

Glycogen Synthase Kinase-3 (GSK3) activity is repressed following insulin treatment of cells. Pharmacological inhibition of GSK3 mimics the effect of insulin on Phospho*eno*lpyruvate Carboxykinase (PEPCK), Glucose-6 Phosphatase (G6Pase) and IGF binding protein-1 (IGFBP1) gene expression. CAAT/enhancer binding protein alpha (C/EBPα) regulates these gene promoters in liver and is phosphorylated on two residues (T222/T226) by GSK3, although the functional outcome of the phosphorylation has not been established. We aimed to establish whether CEBPα is a link between GSK3 and these gene promoters.

**Results:**

C/EBPα represses the IGFBP1 thymine-rich insulin response element (TIRE), but mutation of T222 or T226 of C/EBPα to non-phosphorylatable alanines has no effect on C/EBPα activity in liver cells (towards the TIRE or a consensus C/EBP binding sequence). Phosphorylation of T222/T226 is decreased by GSK3 inhibition, suggesting GSK3 does phosphorylate T222/226 in intact cells. However, phosphorylation was not altered by treatment of liver cells with insulin. Meanwhile C/EBPα activity in 3T3 L1 preadipocytes was enhanced by mutation of T222/T226 and/or S230 to alanine residues. Finally, we demonstrate that C/EBPα is a very poor substrate for GSK3 *in vitro *and in cells.

**Conclusion:**

The work demonstrates an important role for this domain in the regulation of C/EBPα activity in adipocytes but not hepatocytes, however GSK3 phosphorylation of these residues does not mediate regulation of this C/EBP activity. In short, we find no evidence that C/EBPα activity is regulated by direct phosphorylation by GSK3.

## Background

Glycogen synthase kinase-3 (GSK3), an insulin-inhibited protein kinase, has been linked to the control of many cellular processes [1]. It was originally identified as the protein kinase that phosphorylated and inactivated glycogen synthase in rabbit muscle [2]. Two highly related forms of GSK3 (GSK3α and GSK3β) are expressed from distinct genes [3,4]. They share greater than 95% identity in their kinase domains and appear to be ubiquitously expressed in mammals [3,4]. In resting cells GSK3 activity is high and inhibition of the kinase is achieved by at least two mechanisms, firstly through phosphorylation of an N-terminal serine residue (Ser-21 in GSK3α, Ser-9 in GSK3β) [5,6], and secondly through protein protein interaction [7]. Insulin promotes the phosphorylation of Ser-9/21 of GSK3 by activation of protein kinase B (PKB, also known as c-AKT) [8], while canonical wnt signalling inhibits GSK3 independently of this N-terminal phosphorylation [9].

In liver, inhibition of GSK3 reduces expression of the Phosphoenolpyruvate Carboxykinase (PEPCK) and Glucose-6 Phosphatase (G6Pase) genes, two rate limiting enzymes in hepatic glucose production [10], as well as the IGF binding protein-1 (IGFBP1) gene [11]. These gene promoters share a related DNA sequence termed the thymine-rich insulin response element (TIRE) that plays an important role in their response to insulin [12-14]. Expression of these genes as well as GSK3 activity is abnormally high in insulin resistant states such as Type 2 diabetes mellitus (T2DM) [15-19]. Meanwhile overexpression of GSK3 antagonises insulin regulation of IGFBP1 [11]. Therefore inhibition of GSK3 is proposed as a potential target for the treatment of T2DM [1,20,21]. Indeed selective GSK3 inhibitors significantly reduce hepatic glucose output and blood glucose levels in several animal models of obesity and diabetes [22-25]. However, GSK3 is reported to have over 30 substrates, and mutation of one of these, APC, is linked to the development of colonic cancer [20,21,26]. In addition, genetic deletion of one of the isoforms (GSK3β) leads to liver cell death, possibly due to increased sensitivity to TNFα [27]. Hence great care and consideration is being given to the development of GSK3 inhibitors for use in humans. With this in mind we have initiated a search for molecules that link GSK3 to the gluconeogenic gene promoters and in particular the TIRE, since inhibition of GSK3 is sufficient to repress the isolated DNA element [11]. Identification of the GSK3 substrate(s) that regulates IGFBP1, G6Pase and PEPCK gene expression, may permit manipulation of this specific interaction without affecting other GSK3 targets, thereby reducing potential side effects. GSK3 is an unusual kinase in that most substrates must be phosphorylated (primed) on a Ser/Thr four or five residues C-terminal to the GSK3 target residue (see [1] for review). Priming of the substrate is performed by a distinct protein kinase, therefore inhibition of the priming kinase indirectly inhibits GSK3 regulation of the substrate, but only those substrates primed by that particular priming kinase. Priming kinases are potential targets for more specific interference with GSK3 function.

The basic leucine zipper transcription factor, CAAT-enhancer binding protein alpha (C/EBPα) is a potential link between GSK3 and the gluconeogenic genes based on the following information in the literature; 1) C/EBPα is phosphorylated on two residues (T222 and T226, numbers refer to rodent sequence) by GSK3, although the functional effect of phosphorylation is not yet clear [28], 2) C/EBPα is a key molecule in the regulation of the PEPCK and G6Pase gene promoters by cAMP [29-33] and T3 [34], 3) C/EBPα is regulated by wnt [35] and insulin [36,37] in brown adipocytes, and the insulin regulation involves PKB (a regulator of GSK3) [37], 4) PEPCK and G6Pase expression, as well as glucose and glycogen metabolism is abnormal in animals lacking C/EBPα [38-40] and 5) binding of a TIRE-interacting protein is blocked using an oligonucleotide representing a known C/EBPα binding sequence [41].

Therefore, we attempted to determine whether C/EBPα could modulate the activity of the isolated TIRE, or the gene promoters of interest, in a GSK3 regulated manner. In addition, although T222/T226 of C/EBPα were identified as targets for GSK3 it has not previously been established whether S230 was a target for a 'priming' kinase, so we have also investigated whether mutation of S230 influences C/EBPα regulation.

## Results and discussion

### C/EBPα regulates the TIRE independently of T222/226 phosphorylation

**Figure 1:**
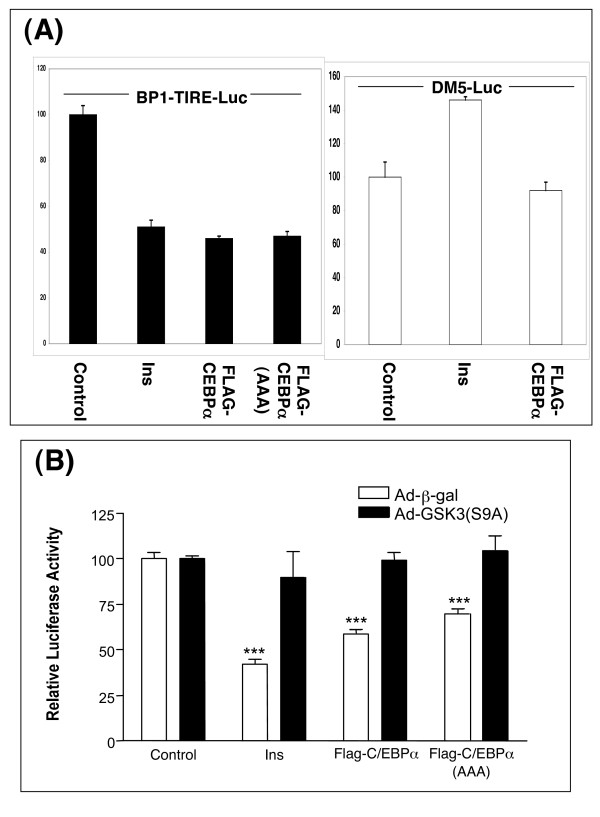
**C/EBPα expression suppresses the BPl-TIRE-Luc reporter activity in H4IIE cells, independently of the phosphorylation status of T222/T226**. (A) H4IIE cells were transfected with BPl-TIRE-Luc (10 μg) or DM5-Luc (10 μg) along with either pcDNA6 vector (1 μg), Flag-C/EBPα (1 μg) or Flag-C/EBPα-AAA (1 μg). Subsequently, cells were incubated with or without 10 nM insulin for 20 h prior to lysis and luciferase assay. Results are presented as relative luciferase activity to the control (serum free) and are average ± SEM (n = 3) from two independent experiments. (B) H4IIE cells were infected with adenovirus expressing either β-Galactosidase (AD-βgal) or active GSK3β (Ad-GSK3 S9A) for 20 h prior to transfection as described in (A). Results are presented as relative luciferase activity to the control (serum free) and are average ± SEM (n = 6) from two independent experiments. *** p < 0.001 compared to control (ad-b-gal).

We have previously shown that insulin, or pharmacological inhibition of GSK3, reduced the expression of endogenous PEPCK, G6Pase and IGFBP1 genes and also the activity of the isolated thymine-rich insulin response element (TIRE) found in these gene promoters [10,11]. In an attempt to further characterise the GSK3 regulation of PEPCK, G6Pase and IGFBP1 we assessed whether a target for GSK3, namely C/EBPα, could regulate TIRE activity. In the hepatocyte C/EBPα activity is linked to cAMP induction of genes, so insulin inhibition of C/EBPα could explain repression of these genes by insulin. In this model, phosphorylation of C/EBPα by GSK3 would have to induce C/EBPα activity toward the TIRE. Interestingly, C/EBPα overexpression had a repressive effect on TIRE activity in the rat hepatoma H4IIE cells (Fig 1A), and the repression was lost when the TIRE sequence was mutated at two residues to produce an insulin insensitive sequence (DM5 [42]; Fig 1A). Hence C/EBPα regulated the TIRE in a sequence specific and insulin-like manner. However, mutation of the GSK3-regulated residues on C/EBPα (T222/T226/S230) to alanines (C/EBPα-AAA) did not alter the ability of C/EBPα to repress TIRE activity (Fig 1A). One possibility is that the dephosphorylated form of C/EBPα *repressed *the TIRE, and that the overexpressed C/EBPα was not substantially phosphorylated by GSK3. Consistent with this hypothesis, co-expression of an active form of GSK3 reduced the effect of wild-type C/EBPα on the TIRE (Fig 1B). Unfortunately, increased GSK3 activity also blocked the repressive action of the C/EBPα-AAA (Fig 1B) clearly demonstrating that phosphorylation of T222/T226/S230 was not required for the action of GSK3 on the isolated TIRE in H4IIE cells. This data questioned the functional importance of T222/T226 phosphorylation in the regulation of C/EBPα activity.

### GSK3 phosphorylates C/EBPα in intact cells

**Figure 2:**
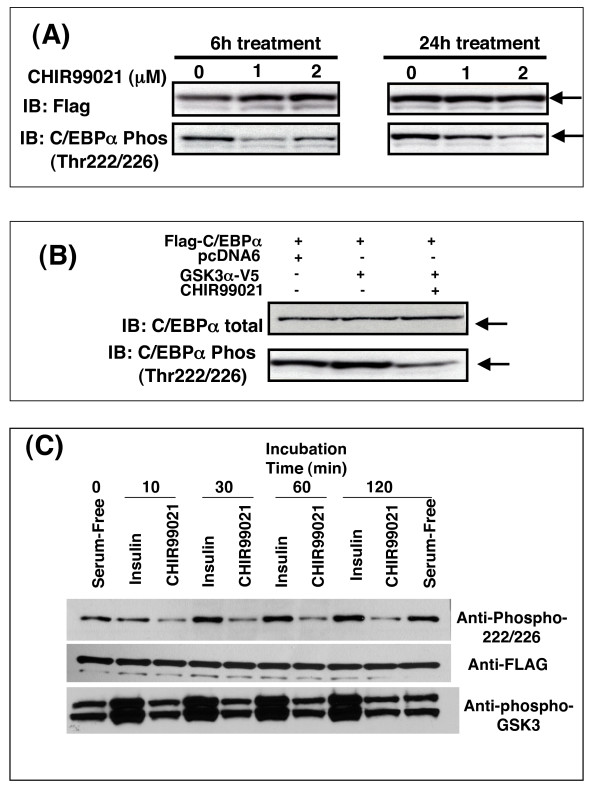
**C/EBPα phosphorylation in intact cells**. AD293 cells were transiently transfected with Flag-C/EBPα (10 μg) prior to incubation with CHIR99021 as indicated (A). Cell lysates were then subjected to Western Blotting with the antibodies as labelled. A representative immunoblot (of 2 two separate experiments) is given. (B) AD293 cells were transfected with Flag-C/EBPα (10 μg) plus GSK3α-V5 (10 μg) or pcDNA6 (10 μg) prior to 24 h incubation with CHIR99021 (2 μM), lysis and Western Blot analysis. A representative immunoblot (of three separate experiments) is given. (C) H4IIE cells were infected with adenovirus expressing Flag-C/EBPα. After 16 h the infected cells were serum starved for 3 h prior to incubation with or without insulin (10 nM) or CHIR99021 (2 μM) for the times indicated. Cell lysates were immunoblotted with the antibodies indicated and a representative experiment is provided.

**Figure 3:**
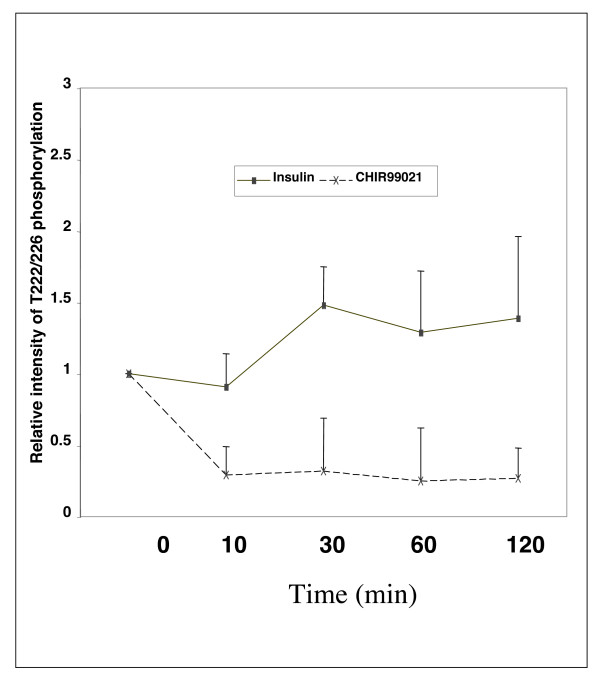
**Quantification of three experiments performed as in Fig 2C**. Results are the average ± SEM.

It was therefore important to establish whether T222 and T226 were actually phosphorylated by GSK3 in intact cells. Cells were transfected with wild-type and mutant C/EBPα and total and phosphorylated C/EBPα visualised by Western Blotting. T222/226 phosphorylation, measured with this antibody, was clearly present in intact cells (Fig 2). Incubation with 1 or 2 μM of the GSK3 inhibitor CHIR99021 [11,43] for 6 or 24 h significantly reduced the phosphorylation of T222/226 of wild-type C/EBPα (Fig 2A), confirming that GSK3 was a T222/226 kinase in these cells. At shorter incubations, 1 μM CHIR99021 was sufficient to give maximal reduction in T222/226 phosphorylation, while 2 μM had a greater effect at 24 h incubation. This is similar to the effect of these concentrations on β-catenin activation in the H4IIE cells [11]. Meanwhile, co-expression of active GSK3α (Fig 2B) only produced a marginal induction of phosphorylation (around 1.2 fold) suggesting that the expressed C/EBPα was either almost fully phosphorylated at T222/226 or that it was not a particularly good substrate for GSK3. Mutation of T222/226 to alanines made the mutant C/EBPα unresponsive to the phosphospecific antibody (see later). Interestingly, the dephosphorylation of C/EBPα in H4IIE cells treated with CHIR99021 was rapid, but not complete (Fig 2C). However, there was no significant reduction in T222/226 phosphorylation following up to 2 h incubation with insulin (Fig 2C and Fig 3), even though insulin promoted phosphorylation of Ser9/Ser21 of GSK3β and GSK3α respectively. Insulin reduced GSK3 activity around 50% in the H4IIE cell line but this is clearly not sufficient to reduce T222/226 phosphorylation. Therefore it appears that insulin does not regulate T222/226 phosphorylation in these cells through inhibition of GSK3. However, the data suggested that some level of T222/226 phosphorylation by GSK3 occurs on the overexpressed protein (Fig 2C).

### C/EBPα is a relatively poor substrate for GSK3 in vitro

**Figure 4:**
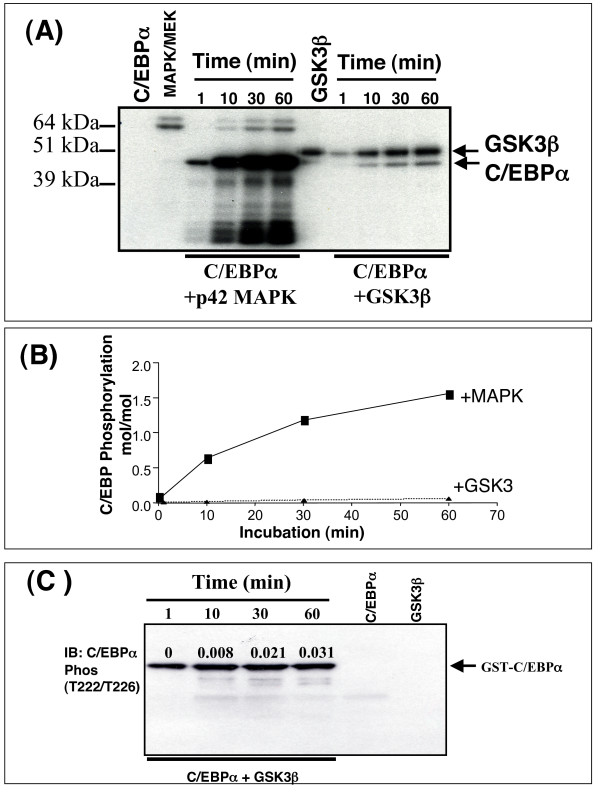
**C/EBPα phosphorylation *in vitro***. (A) Recombinant Flag-C/EBPα (6 pmol) was generated by scission of the GST-C/EBPα fusion protein and incubated with either recombinant GSK3β (2 U/ml) or p42 MAPK (10 U/ml) and [γ-32P]-ATP for 1, 10, 30, and 60 min. The reaction was stopped and separated by SDS-PAGE. (B) The stoichiometry of Flag-C/EBPα phosphorylation in (*A*) by either MAPK or GSK3β is quantified and presented. (C) GST-C/EBPα (7.5 pmol) was incubated with recombinant GSK3β (2 U/ml) and ATP for 1, 10, 30, and 60 min. The reaction was stopped and separated by SDS-PAGE and subjected to Western Blot analysis for phospho-T222/226. The equivalent stoichiometry of phosphorylation (mol/mol) calculated in A is indicated above each band.

We next characterised C/EBPα phosphorylation by GSK3 *in vitro *(Fig 4). Incubation of C/EBPα with GSK3β for up to one hour resulted in a slow incorporation of less than 0.03 mol phosphate per mol of protein (Fig 4A and 4B). In comparison, p42MAP kinase phosphorylation of C/EBPα increased over 60 min peaking around 1.5 mol per mol (Fig 4A and 4B). Phosphorylation of C/EBPα by p42MAP kinase did not occur at T222, T226 or S230 (see below) and is more likely to be on S21 [44]. Interestingly western blot analysis of C/EBPα following incubation with GSK3β gave little indication of this slow increase in phosphorylation, and a very strong signal was obtained with the phosphospecific antibody even at low stoichiometry of phosphorylation (Fig 4C). These data demonstrated that C/EBPα was a relatively poor substrate for GSK3 *in vitro *,but that the phospho-T222/226 antibody was highly sensitive. It is difficult to directly compare phosphorylation of C/EBPα with other substrates of GSK3 as most are poorly phosphorylated prior to priming. For example CRMP phosphorylation by GSK3 (under identical conditions to those used for C/EBPα in Fig 4) incorporates around 0.2 mol/mol phosphate within 2 h of incubation [45]. However, this increases more than 5-fold if the substrate is first primed. Of course we can never rule out the possibility that an intrinsic factor improves the GSK3-C/EBPα interaction *in vivo *,either promoting priming at an unidentified residue or allowing greater phosphorylation by GSK3.

### GSK3 phosphorylation of T222/226 in cells does not require S230 phosphorylation

**Figure 5:**
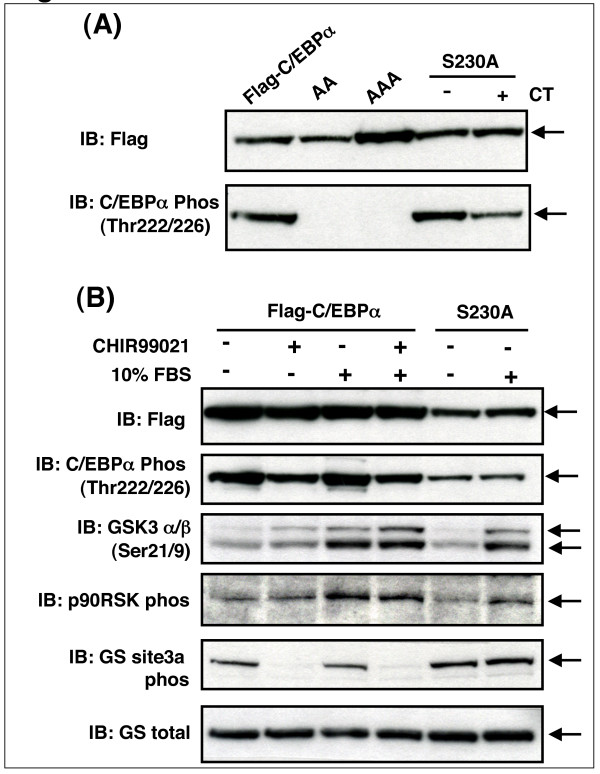
**S230 phosphorylation does not prime for T226 phosphorylation by GSK3**. (A) AD293 cells were transfected with 10 μg of Flag-C/EBPα, Flag-C/EBPα-AA, Flag-C/EBPα-AAA or Flag-C/EBPα-S230A prior to 24 h incubation with 2 μM CHIR99021 (CT), lysis and Western Blot analysis. A representative immunoblot of two such experiments is given. (B) AD293 cells were transfected with 10 μg of Flag-C/EBPα or Flag-C/EBPα-S230A, then serum starved overnight prior to a 2 h incubation with CHIR99021 (2 μM) or 10%(v/v) FBS. Cell lysates were then subjected to Western Blotting with the antibodies as labelled. A representative immunoblot of two such experiments is given.

Many substrates of GSK3 are primed for kinase recognition by prior phosphorylation at a S/T residue four amino acids C-terminal to the target site [1]. S230 lies within this 'consensus' GSK3 targeting sequence (Table 2). Therefore S230 phosphorylation could prime for T226 phosphorylation by GSK3, permitting subsequent phosphorylation of T222 by GSK3. However, mutation of S230 to alanine had no effect on T222/226 phosphorylation in intact cells (Fig 5). This argued that S230 phosphorylation did not prime for T226 phosphorylation by GSK3 in resting cells, or that the wild-type C/EBPα is not being efficiently phosphorylated at S230. Since the amino acid immediately following S230 is a proline (Table 2), we hypothesised that priming may only occur after induction of a proline-directed kinase such as a member of the MAP kinase family. Therefore we attempted to induce S230 phosphorylation by incubation of cells with serum, which stimulates the classical MAP kinase and several other proline-directed kinases such as cyclin-dependent kinases. However, T222/226 phosphorylation of wild-type or S230A C/EBPα was not altered in serum treated cells (Fig 5B), despite activation of the p90RSK downstream of the p42/p44MAP kinase (Fig 5B). This demonstrated we could not alter S230 phosphorylation by incubating cells with or without serum. Interestingly, serum treatment induced phosphorylation of S21/9 of GSK3α/β (Fig 5B), suggesting some inhibition of GSK3 occurs in the serum treated cells. This was not sufficient to reduce C/EBPα phosphorylation but did partially reduce glycogen synthase phosphorylation (a known substrate of GSK3 ([46]; Fig 5B). Similarly, treatment of the cells with CHIR99021 did not completely inhibit T222/226 phosphorylation (Fig 2 and Fig 3), despite a complete loss of glycogen synthase phosphorylation (Fig 5B). Therefore T222/226 must be relatively resistant to dephosphorylation or a distinct T222/226 protein kinase must be present in these cells. Again, potential substrates for GSK3 have been reported that have their priming at a residue more distant from the GSK3 target site, and we cannot discount that possibility. However, our data strongly argues against priming through S230 phosphorylation.

**Figure 6:**
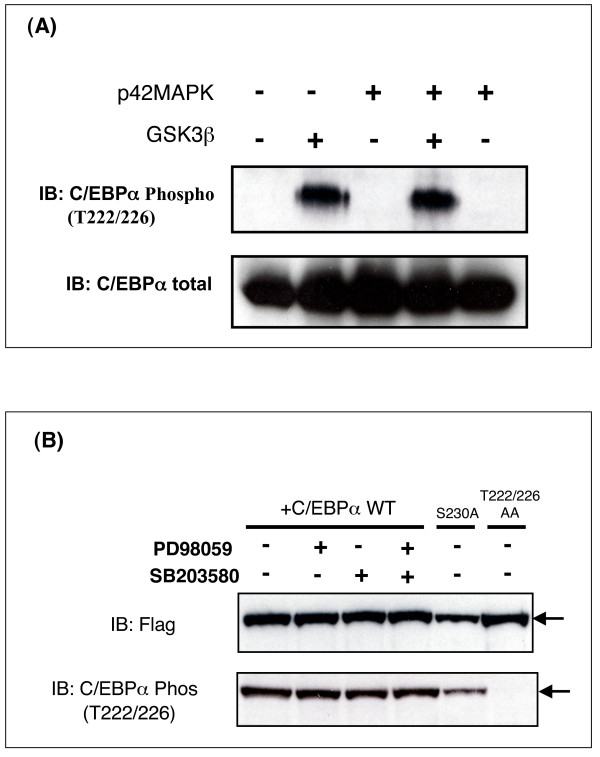
**MAPK does not influence CEBPα phosphorylation by GSK3**. (A) Recombinant Flag-C/EBPα (6 pmol) was incubated for 60 min with or without p42 MAPK (10 U/ml) and/or GSK3β (2 U/ml) and Mg ATP, as indicated in figure. The reaction was stopped and separated by SDS-PAGE and subjected to Western Blot. (B) AD293 cells were transfected with 10 μg of Flag-C/EBPα, Flag-C/EBPα-AA, or Flag-C/EBPα-S230A prior to 24 h incubation with 10 μM PD98059, SB203580 or DMSO, lysis and Western Blot analysis. A representative immunoblot of two such experiments is given.

Prephosphorylation of CEBPα by p42 MAP kinase did not prime for subsequent phosphorylation by GSK3 *in vitro *(Fig 6A), confirming that S230 is not targeted by p42 MAP kinase. Similarly, incubation of cells with either a p42MAP kinase inhibitor (PD98059) or a p38MAP kinase inhibitor (SB203580) had no effect on phosphorylation of CEBPα at T222/226 (Fig 6B).

### C/EBPα induced a consensus binding element while repressing the TIRE in H4IIE cells

**Figure 7:**
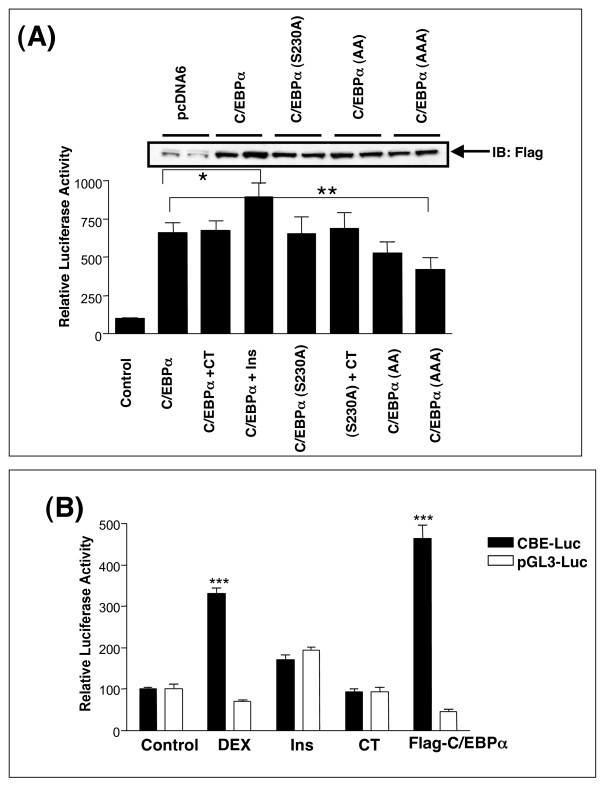
**C/EBPα activity in H4IIE cells**. (A) H4IIE cells were transfected with the CBE-Luc (10 μg) along with 1 μg of pcDNA6, Flag-C/EBPα, Flag-C/EBPα-AA, Flag-C/EBPα-AAA or Flag-C/EBPα-S230A prior to a 20 h incubation with insulin (10 nM) or CHIR99021 (CT, 2 μM). Results are presented as relative luciferase activity to control (serum free) and are average ± SEM (n = 6) from two independent experiments. * p = 0.0683, ** p = 0.0523. (B) H4IIE cells were co-transfected with 10 μg of either CBE-Luc reporter or pGL3-Luc reporter plus 1 μg of pcDNA6 or Flag-C/EBPα prior to 20 h incubation with dexamethasone (500 nM), insulin (10 nM), or CHIR99021 (CT, 2 μM). Results are presented as relative luciferase activity to the control (serum free) and are average ± SEM (n = 6) from two independent experiments. ***p < 0.001

In contrast to the repressive action on TIRE activity, overexpression of C/EBPα in H4IIE cells induced the activity of a consensus C/EBP binding element (CBE) (Fig 7A). We were therefore able to investigate whether the GSK3 regulation of T222/T226 was dependent on the DNA sequence analysed. Mutation of T222/226/S230 to alanines (in any combination) made no difference to the induction of the CBE by C/EBPα (Fig 7A). Similarly, inhibition of GSK3 using CHIR99021 or insulin had no effect on CBE activity (Fig 7A and 7B). Therefore GSK3 phosphorylation of T222/226 does not regulate either induction of the CBE or repression of the TIRE in the H4IIE cells. Interestingly CBE activity was induced in the presence of the synthetic glucocorticoid dexamethasone to a similar level as that seen with C/EBPα overexpression (Fig 7B).

### C/EBPα overexpression does not regulate endogenous TIRE-containing genes

**Figure 8:**
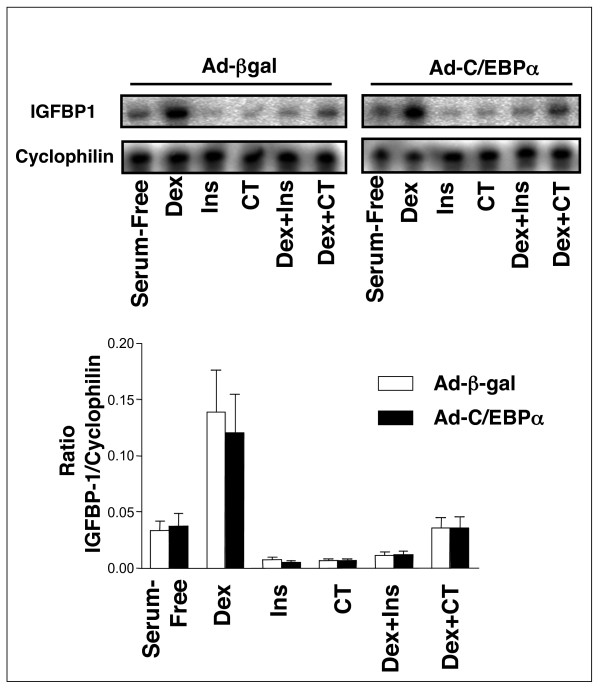
**Regulation of endogenous IGFBP1 gene expression in H4IIE cells following C/EBPα overexpression**. H4IIE cells were infected with adenovirus expressing either β-Galactosidase (AD-βgal) or Flag-C/EBPα (Ad-C/EBPα). Infected cells were serum starved overnight prior to a 3 h incubation with insulin (10 nM), dexamethasone (500 nM), CHIR99021 (CT, 2 μM), or a combination of each as indicated. RNA was extracted and IGFBP1 expression was measured by RPA. Expression was calculated relative to cyclophilin mRNA levels. Data are presented as the average ± SEM of three experiments, with a representative autoradiograph shown in the upper panels.

**Figure 9:**
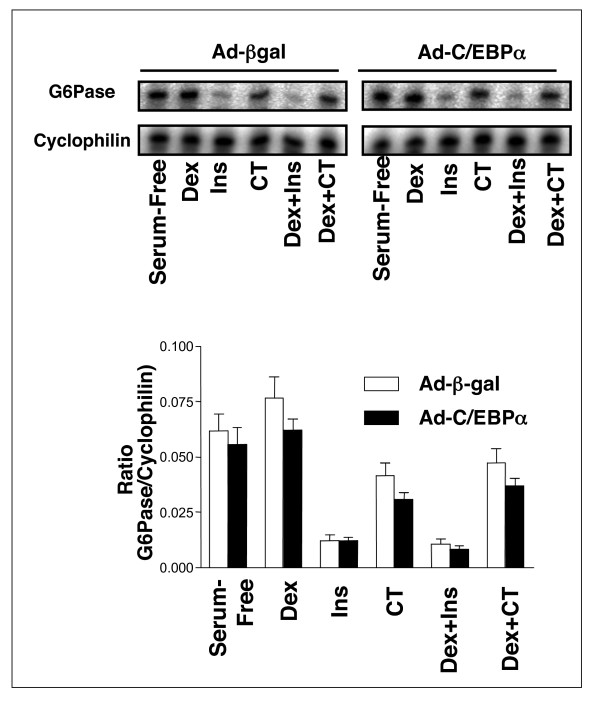
**Regulation of endogenous G6Pase gene expression in H4IIE cells following C/EBPα overexpression**. RNA produced as in Fig. 8 was analysed for G6Pase expression by RPA. Expression was calculated relative to cyclophilin mRNA levels. Data are presented as the average ± SEM of three experiments, with a representative autoradiograph shown in the upper panels.

**Figure 10:**
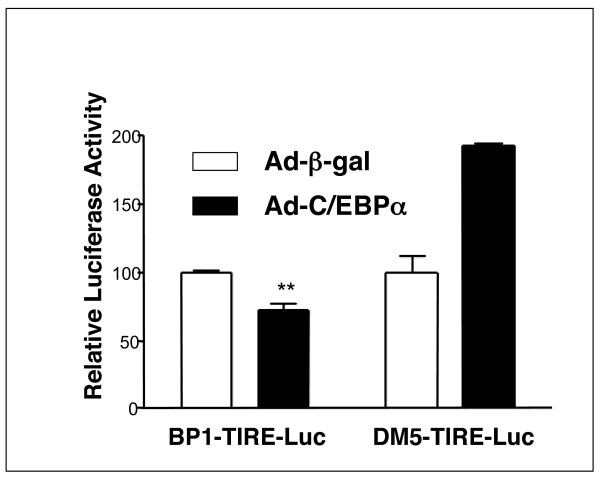
**Regulation of the isolated TIRE in H4IIE cells following C/EBPα overexpression**. H4IIE cells, infected as in Fig. 8, were also transfected with 10 μg of BPl-TIRE-Luc or DM5-Luc and luciferase activity measured 20 h later. Results are presented as relative luciferase activity to the control (AD-βgal, serum free) and are average ± SEM of three experiments. ** p < 0.01 AD-βgal versus Ad-C/EBPα.

In order to establish whether changes in C/EBPα phosphorylation could alter expression of the insulin regulated genes IGFBP1 and G6Pase, we overexpressed C/EBPα in H4IIE cells using an adenoviral vector to produce almost 100% transfection efficiency (Figs 8, 9, 10). We observed no effect of C/EBPα expression on IGFBP1 (Fig 8) or G6Pase (Fig 9) gene expression, and no alteration in regulation of either gene by dexamethasone, insulin or CHIR99021. However, there was a significant repression of the co-expressed BP1-TIRE by C/EBPα when compared with its effect on the insulin resistant TIRE (DM5) (Fig 10). This suggests that C/EBPα overexpression represses the isolated element but has no effect on this element when it is in the context of the intact gene promoters. This is an example of the dangers of studying isolated promoter elements and extrapolating to intact gene promoters without examining endogenous regulation. Interestingly, a postnatal knockout of C/EBPα resulted in reduced basal expression of PEPCK and G6Pase but did not alter regulation of these genes by cAMP [40]. Again, this would argue against a repressive effect of this factor on the endogenous genes.

### C/EBPα regulation of the CBE in preadipocytes is greater when T222/226 are mutated

**Figure 11:**
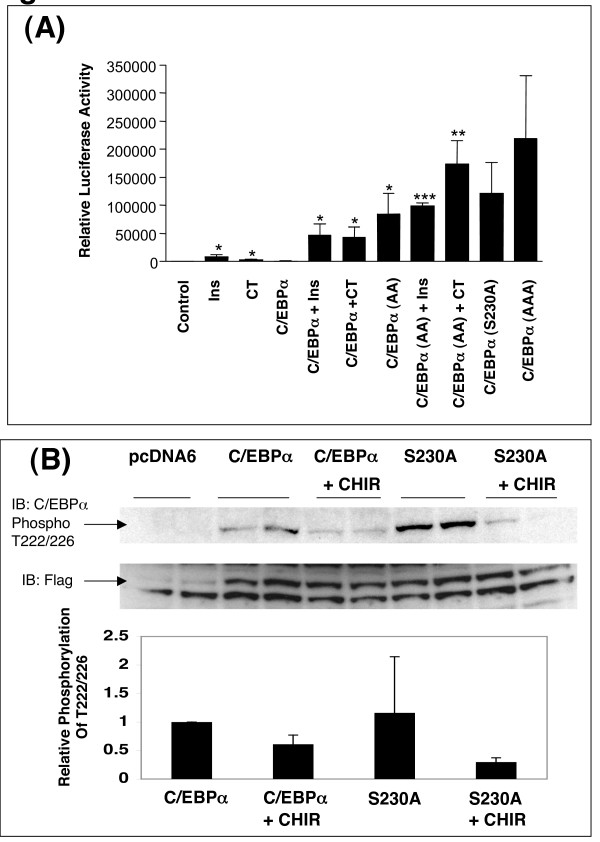
**Mutation of T222/226 and S230 alters C/EBPα activity in preadipocytes**. A) 3T3L1 preadipocytes were transfected with 2 μg of C/EBP-Luc reporter along with 200 ng of pcDNA6, Flag-C/EBPα, AA, AAA, or S230A as described in the material and methods. Cells were incubated with insulin (l00 nM) or CHIR99021 (CT, l0 μM) for 20 hr prior to harvest for luciferase assay. Results are relative luciferase activity and are average ± SEM (n = 6) from two independent experiments. * p < 0.05, ** p < 0.01, *** p < 0.005 compared with control. B) 3T3L1 preadipocytes were transfected with 200 ng of pcDNA6, Flag-C/EBPα wild type, or Flag-C/EBPα S230A, ± 2 μM CHIR99021 (CT). Cells were lysed, the recombinant protein immunoprecipitated using anti-FLAG, and visualised using anti-FLAG or anti-phospho222/226. A representative experiment is shown in the upper panel, while a quantification of three such experiments is given in the lower panel.

C/EBPα has an important role in adipocyte differentiation [37,47,48], therefore we transfected 3T3-L1 preadipocytes with C/EBPα and measured CBE activity (Fig 11A). In contrast to the H4IIE cells, mutation of T222/226 to alanines resulted in a significant increase in transactivation potential compared to wild-type C/EBPα (Fig 11A). This suggested that phosphorylation of these residues was inhibiting C/EBPα activity in these cells. Consistent with this hypothesis, inhibition of GSK3 using CHIR99021 or insulin induced wild-type C/EBPα activity (Fig 11A). In addition, the C/EBPα S230A mutant had similar transactivation potential to the AA mutant (Fig 11A), strongly suggesting that S230 was a priming site for T222/T226 phosphorylation in these cells. However we could find no evidence that phosphorylation of T222/226 was reduced in the S230A mutant (Fig 11B). Although there was a great deal of inter-experiment variation in the phosphorylation of T222/T226 in the WT and the S230A mutant there was not a significant difference between the two, and in 2 out of the three experiments the S230A mutant exhibited higher phosphorylation than the WT protein (Fig 11B). Therefore, changes in phosphorylation of these two sites cannot account for the difference in C/EBPα activity when the S230 site is mutated to alanine. Ideally it would be beneficial to confirm these data on endogenous gene expression in the adipocyte cell line, however these cells do not transfect efficiently with the adenoviral constructs making it impossible to examine their effect on endogenous genes.

## Conclusion

In summary, C/EBPα is not the link between GSK3 and the hepatic TIRE-containing gene promoters. However, C/EBPα can regulate the TIRE, at least when overexpressed, and the residues T222/226 and S230 all influence C/EBPα function in preadipocytes. Interestingly we demonstrate opposite effects of C/EBPα overexpression on TIRE and CBE activity, and a tissue specific role for the T222/226 motif in this activity. Our data also provides further evidence of the differences in transcription factor activity when measured using isolated promoter elements as opposed to intact gene promoters. During the course of this study a regulation of C/EBPβ by GSK3 in adipocytes was reported [49]. Therefore it will be of interest to confirm whether this C/EBP isoform is the link between GSK3 inhibition and TIRE regulation in hepatocytes.

## Methods

### Material

Radioisotopes [γ-^32^P]ATP (Amersham Biosciences, Inc., Little Chalfont, Buckinghamshire, UK) and [α-^32^P]UTP (ICN, Thame, Oxfordshire, UK) were purchased from the indicated sources. Insulin was obtained from Novo Nordisk (Crawley, West Sussex, UK), dexamethasone and IBMX (Sigma, Poole, Dorset, UK), RNAse Protection Assay Kit II was from AMS Biotech/Ambion (Austin, TX, USA). CHIR99021 was synthesised by Dr Rudolpho Marquez [11]. Antibodies to the following epitopes were purchased from the companies in parenthesis; C/EBPα (Santa Cruz Biotechnologies, UK), phospho-C/EBPα (T222/226) and phospho-GSK3α/β (S21/9) (Cell Signaling Technologies, Hertfordshire, UK), Flag (Sigma, Poole, Dorset, UK), while the Glycogen Synthase (total and site 3 phospho antibody), and p90 RSK phospho antibody were made in the Division of Signal Transduction Therapy, University of Dundee.

### Cell culture

The rat hepatoma cell line H4IIE was cultured in Dulbecco's Modified Eagle's Medium (DMEM) containing 1000 mg/L glucose, 5% (v/v) foetal calf serum (GIBCO, Carlsbad, CA). The AD293 cell line was maintained in DMEM containing 4500 mg/L glucose, 10% (v/v) foetal calf serum, while 3T3-L1 preadipocytes were grown in DMEM containing 4500 mg/L glucose and 10% (v/v) newborn calf serum (GIBCO).

### Construction of plasmid DNA, luciferase reporter, and mutagenesis

The full length C/EBPα cDNA was amplified by PCR from a rat L6 cDNA library and BamHI/Kosac/Flag and EcoRl sequences introduced at the 5' and 3' end respectively as indicated in Table 1. The PCR product was subcloned into TOPO one shot (Invitrogen), verified by DNA sequencing, and subcloned into pcDNA6, GST-pGEX-6, and pshuttle-CMV vectors for the purpose of expression in mammalian cells, production of recombinant GST-tagged protein in *Escherichia coli *BL21 cells, and construction of recombinant adenovirus (AdEasy XL Adenoviral Vector System, Stratagene), respectively. Flag-C/EBPα point mutants, S230A, T222A/T226A (AA), and T222A/T226A/S230A (AAA) were generated using the QuickChange mutagenesis kit (Stratagene) with the oligo sequences shown in Table 1. The AAA mutant was generated by introducing the S230A point mutation into the AA mutant. A luciferase reporter construct containing a C/EBP consensus binding sequence (CBE-Luc) 5' of a thymidine kinase promoter was constructed using the oligos shown in Table 1. Both sense and antisense oligos were annealed prior to restriction digest with Kpnl and Bgl II. The product was subcloned into the BP1 luciferase reporter linearised with Kpnl and BamHI to remove the TIRE (the luciferase construct was a gift from Dr Robert Hall and Professor Daryl K. Granner (Vanderbilt University, TN, USA)).

### Transient transfection

AD293 cells were transiently transfected with 10 μg of pcDNA6, Flag-C/EBPα, AA, AAA, or S230A mutants by the calcium phosphate method. DNA precipitant was added to cells for 4 h, prior to addition of inhibitors and hormones at the concentrations and for the times indicated in figure legends.

H4IIE cells were transfected as described previously [50]. Briefly, 10 μg of BP1-TIRE-Luc, the insulin insensitive point mutant of the TIRE (DM5-Luc), C/EBP-Luc, or pGL3-Luc (control), along with 1 μg of pcDNA6, wild type or mutant Flag-C/EBPα constructs were precipitated and incubated with H4IIE cells in suspension. Cells were plated onto 10 cm dishes, allowed to attach for 4 h, prior to DMSO shock and incubation with hormones/inhibitors for 20 h as indicated in figure legends. Cells were lysed in 200 μl lysis buffer (Promega, UK), the cell debris removed by centrifugation at 13,000 × g for 10 min at 4°C, and the supernatant stored at -70°C. Luciferase assays were performed using the firefly luciferase assay system (Promega, UK), according to manufacturer's instructions, with luciferase activity being corrected for the protein concentration in the cell lysate.

3T3-L1 preadipocytes were transfected using Fugene 6 Transfection Reagent (Roche) following manufacture's protocol. C/EBP-Luc (2 μg) along with 200 ng of pcDNA6, wild type or mutant Flag-C/EBPα constructs was complexed with Fugene 6 transfection reagent prediluted in Serum Free DMEM at the ratio of 1:3 (μg DNA : μl Fugene 6) for 20 min at RT before addition to cells. After 4 h at 37°C an equal amount of DMEM containing 20% foetal calf serum was added. 48 h post-transfection, cells were washed with serum free DMEM and incubated for a further 24 h in serum free media with or without hormones or inhibitors as described in figure legends. Cells were lysed in 100 μl lysis buffer and luciferase activity determined as above.

We estimate that the level of over expression of C/EBPα is roughly 4 fold in the adipocytes, around 6-fold in the liver cells by transient transfection and nearly 10 fold in adenovirally infected H4IIE experiments

### In vitro phosphorylation of C/EBPα

Purified recombinant C/EBPα (6 pmol) was incubated at 30°C with His_6_-GSK3β (2 U/ml) or p42 MAPK (10 U/ml), 10 mM magnesium acetate, 0.1 mM [γ-^32^P]ATP (450,000 cpm/nmol) in buffer containing 50 mM Tris-HCl, pH 7.5, 0.03% (v/v) Brij 35, and 0.1% (v/v) 2-mercaptoethanol, for times indicated in the figure legends. Reactions were stopped by the addition of LDS containing sample buffer (Novex), heated at 70°C for 10 min and subjected to SDS-PAGE. The gel was dried on the 3 M Filterpaper, and radioactivity incorporated into recombinant C/EBPα visualized by autoradiography and quantified by phosphorimager (Fuji). A standard curve of radiolabelled ATP was used to assess nmoles of phosphate incorporated into the C/EBPα substrate. p42 MAP kinase and GSK3 activities were calculated by in vitro phosphorylation of myelin basic protein and P-GS peptide, respectively.

### Preparation of cell extract for SDS PAGE and Immunoblotting

Cells were washed with ice cold PBS twice and scraped into ice-cold lysis buffer (25 mM Tris/HCl, pH 7.4, 50 mM NaF, 100 mM NaCl, 1 mM sodium vanadate, 5 mM EGTA, 1 mM EDTA, 1% (v/v) Triton X-100, 10 mM sodium pyrophosphate, 1 mM benzamidine, 0.1 mM PMSF, 0.27 M sucrose, 2 μM microcystin and 0.1% (v/v) 2-mercaptoethanol). Cell debris was removed by centrifugation at 13,000 × g for 10 min at 4°C. Preparation of nuclear protein extracts was performed using a Nuclear Extraction Kit (Panomics), as per the manufacture's instructions. The protein concentration was determined by Bradford assay (Sigma), using BSA as standard as per the manufacture's instructions. Protein from cell lines (10–20 μg from H4IIE, 50 μg from AD293 or 3T3 preadipocytes) was separated on Novex SDS 4–12% polyacrylamide gels. Following transfer to nitrocellulose, blots were blocked with 5% (w/v) non-fat milk in TBST (Tris-buffered saline containing 0.1% (v/v) Tween 20) for 1 h, and incubated with primary antibodies at 4°C overnight prior to incubation for 1 h at RT with the secondary antibody and development using ECL kit (Amersham Biosciences, Inc.).

### Adenoviral Infection, RNA extraction and RNAse protection assay

For RNA studies, H4IIE cells were infected with recombinant adenovirus expressing Flag-C/EBPα (Ad-C/EBPα) or β-Galactosidase (Ad-β-gal) for 4 h at MOI = 50. 24 h later, infected cells were split into 10 cm dishes and serum starved overnight prior to treatment with hormone/inhibitor for 3 h at the concentrations indicated in the figure legends. Total cellular RNA was isolated using TRI Reagent (Sigma) following the manufacturer's instructions. An RNase Protection Assay (RPA) was performed to determine the relative amounts of IGFBP-1, G6Pase, and cyclophilin mRNA as described previously [50]. Band intensity was quantified on a phosphorimager (Fuji), data calculated as a ratio of IGFBP-1 to cyclophilin mRNA. For phosphorylation studies, H4IIE cells were infected with recombinant adenovirus expressing Flag-C/EBPα (Ad-C/EBPα) at MOI = 100. 16 h later, infected cells were serum starved for 3 h prior to treatment with hormone/inhibitor for the times and at the concentrations indicated in the figure legends.

### Statistics

The comparison of one variable between two groups was determined by unpaired Student's t-test with the aid of PRISM 3.0 (Graphpad Software, USA) and/or Microsoft Excel. The values were expressed as mean ± S.E.M.

## Abbreviations

PEPCK; Phosphoenolpyruvate Carboxykinase, IGFBP1; IGF binding protein-1, G6Pase; glucose-6-phosphatase, RPA; RNAse Protection Assay, TIRE; thymine rich insulin response element, C/EBP; CAAT enhancer binding protein, CBE; C/EBP binding element.

## Authors' contributions

The majority of the molecular work was performed by HKL, with important contributions from SP (adipocyte analysis, and phosphorylation time course), CL (endogenous gene transcription), and DF (adenoviral synthesis and production). Key reagents were produced to high quality by JH and HM. The project was conceived and jointly supervised by HSH and CS.

## Figures and Tables

**Table 1:** Primers and oligonucleotides used in C/EBP mutagenesis. Primers and oligonucleotides

Constructs	DNA sequences
Flag-C/EBPα	Forward: 5'-GGA TCC GCC ACC ATG GAC TAG AAG GAC GAC GAT GAC AAG GAG TCG GCC GAC TTC TAC GAG-3' Reverse: 5'- GAA TTC TCA CGC GCA GTT GCC CAT GGC CTT GAC-3'
S230A Flag-C/EBPα mutant	Sense: 5'-CGC CCG TGC CCG CCC CTC ATC CCG-3' Antisense: 5'-CGG GAT GAG GGG CGG GCA CGG GCG-3'
Flag-C/EBPα mutant(AA)	Sense:5'-GGC CAC CCT GCG CCG CCG CCG GCG CCC GTG CCC AGC CCT CAT CCC-3' Antisense: 5'-GGG ATG AGG GCT GGG CAC GGG CGC CGG CGG CGG CGC AGG GTG GCC-3'
GSK3α-V5	Forward: 5'-GGA TCC GCC ACC ATG AGC GGC GGC GGG CCT T-3' Reverse: 5'-TCT AGA GGA AGA GTT AGT GAG GGT AGG TGT GGCA-3'
CBE-Luc reporter	Sense:5'-CGG GGT ACC CCG ATT TTT GCG CAA TTT TAT TGC GCA ATC AAT ATT GGA AGA TCT TC- 3' Antisense: 5'-GAA GAT CTT CCA ATA TTG ATT GCG CAA TAA AAT TGC GCA AAA ATC GGG GTA CCC CG-3'

**Table 2:** Comparison of mouse, rat and human C/EBPα sequences surrounding potential GSK3 target residues. Numbers refer to mouse (upper) and human (lower) potential phosphorylated residues. C/EBPα sequences.

	222 226 230
Mouse	PGHP**T**PPP**T**PVP**S**PHAA
Rat	PGHP**T**PPP**T**PVP**S**PHPA
Human	PGHP**T**PPP**T**PVP**S**PHAA
	226 230 234
